# MUC4 immunohistochemistry is useful in distinguishing epithelioid mesothelioma from adenocarcinoma and squamous cell carcinoma of the lung

**DOI:** 10.1038/s41598-017-18545-x

**Published:** 2018-01-09

**Authors:** Amany Sayed Mawas, Vishwa Jeet Amatya, Kei Kushitani, Yuichiro Kai, Yoshihiro Miyata, Morihito Okada, Yukio Takeshima

**Affiliations:** 1 Department of Pathology, Hiroshima University Graduate School of Biomedical and Health Sciences, Hiroshima, Japan; 2 Department of Pathology and Clinical Pathology, South Valley University, Qena, Egypt; 3 Department of Surgical Oncology, Research Institute for Radiation Biology and Medicine, Hiroshima University, Hiroshima, Japan

## Abstract

The differential diagnosis of epithelioid mesothelioma from lung adenocarcinoma and squamous cell carcinoma requires the positive and negative immunohistochemical markers of mesothelioma. The IMIG guideline has suggested the use of Calretinin, D2–40, WT1, and CK5/6 as mesothelial markers, TTF-1, Napsin-A, Claudin 4, CEA as lung adenocarcinoma markers p40, p63, CK5/6, MOC-31 as squamous cell markers. However, use of other immunohistochemical markers is still necessary. We evaluated 65 epithelioid mesotheliomas, 60 adenocarcinomas, and 57 squamous cell carcinomas of the lung for MUC4 expression by immunohistochemistry and compared with the previously known immunohistochemical markers. MUC4 expression was not found in any of 65 cases of epithelioid mesothelioma. In contrast, MUC4 expression was observed in 50/60(83.3%) cases of lung adenocarcinoma and 50/56(89.3%) cases of lung squamous cell carcinoma. The negative MUC4 expression showed 100% sensitivity, 86.2% specificity and accuracy rate of 91.2% to differentiate epithelioid mesothelioma from lung carcinoma. The sensitivity, specificity, and accuracy of MUC4 are comparable to that of previously known markers of lung adenocarcinoma and squamous cell carcinoma, namely CEA, Claudin 4 and better than that of MOC-31. In conclusion, MUC4 immunohistochemistry is useful for differentiation of epithelioid mesothelioma from lung carcinoma, either adenocarcinoma or squamous cell carcinoma.

## Introduction

Malignant mesothelioma is a highly aggressive malignant neoplasm with unfavorable prognosis. The mortality from mesothelioma is increasing in Japan, and other developing countries^1^. Some of the non-mesotheliomatous peripheral lung cancer, adenocarcinoma or squamous cell carcinoma, may present as the pleurotropic growth resembling that of mesothelioma^2^. As the prognosis and treatment protocols of epithelioid mesothelioma are different from that of the lung cancer, accurate diagnosis of mesothelioma is crucial.

Mesothelioma is classified into three major histological subtypes: epithelioid, biphasic, and sarcomatoid mesothelioma. Epithelioid mesothelioma shows numerous histological growth pattern^3^. Due to this resemblance of clinical growth pattern and histology of epithelioid mesothelioma with lung cancers, immunohistochemical markers are essential for accurate diagnosis of epithelioid mesothelioma.

Although IMIG (International Mesothelioma Interest Group) recommended Calretinin, D2–40 (podoplanin), WT1 as mesothelioma markers, CEA, TTF-1, Napsin-A, as lung adenocarcinoma markers, and p63, p40, MOC-31 as for lung squamous carcinoma markers^4^, the additional immunohistochemical markers will surely benefit in some unusual cases.

We recently proposed the addition of two immunohistochemical markers, Intelectin-1 and DAB2, as the positive immunohistochemical markers of epithelioid mesothelioma^5^. In this study, we also found the down regulation of many other genes including MUC4 in epithelioid mesothelioma as mentioned in its supplementary data.

Therefore, we evaluated the diagnostic applicability of MUC4 immunohistochemistry for differentiation of epithelioid mesothelioma to lung adenocarcinoma and squamous cell carcinoma.

## Materials and Methods

### Patients and Histologic Samples

Pathological specimens (formalin-fixed paraffin-embedded tissue blocks) of 65 epithelioid mesotheliomas and 60 lung adenocarcinoma and 57 squamous cell carcinoma were obtained from the tissue archives of the Department of Pathology, Hiroshima University. We also reviewed the patient’s clinical details and chest computed tomography findings for confirmation of the tumor localization. The mean age of lung adenocarcinoma patients was 69 with range from 43 to 84 (male 38, female 22), that of lung squamous cell carcinoma was 69 with range from 39 to 86 (male 49, female 7), and that of epithelioid mesothelioma 69 with range from 33–92 (male 61, female 4).

All histological sections were examined and reclassified by three pathologists (VJA, KK, and YT) according to recent WHO classification^6^. Pathologic diagnosis was confirmed by histologic findings and the immunohistochemical marker panel recommended by 2012 International Mesothelioma Interest Group (IMIG) guideline^4^. The collection of tissue specimens for this study was carried out in accordance with the “Ethics Guidelines for Human Genome/Gene Research” enacted by the Japanese Government. Ethical approval was obtained from the institutional ethics review committee (Hiroshima University E-974). All experimental procedures were in accordance with the with ethical guidelines. Samples used were linked-anonymised archival specimens, and individual consent was not required for this research.

### Immunohistochemical Procedures and Evaluation of MUC4 Expression

Immunohistochemistry was performed using 3 μm tissue sections prepared from the best representative formalin-fixed paraffin-embedded blocks of epithelioid mesothelioma, lung adenocarcinoma, and squamous cell carcinoma cases. Immunohistochemical staining was performed using the Ventana Benchmark GX automated immunohistochemical station (Roche Diagnostics, Tokyo, Japan). The antigen retrieval methods and antibodies used in this study are summarized in Table 1. Incubation with the secondary antibody and detection was performed with Ventana ultraView Universal DAB Detection Kit (Roche Diagnostics). Immunoreactivity was evaluated as either negative or positive. Nuclear staining of calretinin, WT1, p40, p63, and TTF-1, cytoplasmic staining of MUC4 and Napsin-A, CK5/6, CEA, membranous staining of podoplanin (clone: D2–40), Epithelial Related Antigen (clone: MOC-31) and claudin 4 were considered as ‘positive’.

**Table 1:** List of antibodies with their clone, commercial source, and reaction conditions.

Antibody to	Clone	Source	Dilution	Antigen Retrieval
MUC4	8G7	Santa Cruz Biotech	1:25	60 min, CC1
Calretinin	SP65	Ventana-Roche	prediluted	30 min, CC1
Podoplanin	D2–40	Nichirei Bioscience	prediluted	60 min, CC1
WT1	6F-H2	Ventana-Roche	1:25	60 min, CC1
CEA	COL-1	Nichirei Bioscience	Prediluted	8 min, CC1
Claudin 4	3E2C1	Life Technologies	1:100	60 min, CC1
TTF-1	8G7G3/q	Dako-Agilent	1:25	30 min, CC1
Napsin-A	MRQ-60	Ventana-Roche	prediluted	60 min, CC1
Epithelial Related Antigen	MOC-31	Dako-Agilent	1:25	10 min, Protease
p63	DAK-p63	Dako-Agilent	1:25	60 min, CC1
CK5/6	D5/16 B4	Dako-Agilent	1:25	60 min, CC1
p40	BC28	Biocare Medical	1:100	60 min, CC1

WT1: Wilm’s tumor 1; CEA: carcinoembryonic antigen; TTF-1: thyroid transcription factor-1; CK: cytokeratin.

Abbreviation: CC1, cell conditioning buffer 1 (Tris-based buffer, pH 8.5 from Ventana-Roche).

Positive Immunoreactivity was semi quantitatively scored as 0 for none to trace, 1+ for up to 10%, 2+ for 10–50%, and 3+ for >50% tumor cells showing positive expression.

Statistical analyses were performed using the Fisher’s exact test for calculation of p-value of positivity of individual markers, Mann Whitney U test for calculation Z-score and the *p*-value of immunohistochemical score of individual markers. Sensitivity, specificity, and accuracy rate are calculated using a 2 × 2 contingency table model.

## Results

### Immunohistochemical Result

The percentages and immunohistochemical scores of MUC4 expression in epithelioid mesothelioma, lung adenocarcinoma, and squamous cell carcinoma and other immunohistochemical markers are shown in Table 2.

**Table 2:** Immunohistochemical Result of Epithelioid Mesothelioma, Lung Adenocarcinoma, and Squamous Cell Carcinoma.

Antibody	Epithelioid Mesothelioma (65 cases)					Lung Adenocarcinoma (60 cases)					Lung Squamous Cell Carcinoma (56 cases)					Z-score!	
	Positive cases (%)	Immunohistochemical Score				Positive cases (%)	Immunohistochemical Score				Positive cases (%)	Immunohistochemical Score				EM vs LAC	EM vs LSC
		0	1+	2+	3+		0	1+	2+	3+		0	1+	2+	3+		
MUC4	0 (0)	65	0	0	0	50 (83.3)	10	20	9	21	50 (89.3)	6	19	10	21	8.0*	8.4*
Calretinin	64 (98.5)	1	5	2	57	17 (28.3)	43	11	6	0	28 (50)	28	13	10	5	9.1*	7.8*
D2–40	63 (96.9)	2	4	5	54	7 (11.7)	53	4	3	0	34 (60.7)	22	7	18	9	9.2*	6.7*
WT1	56 (86.2)	9	17	7	32	0 (0)	60	0	0	0	2 (3.6)	54	2	0	0	9.3*	9.1*
CEA	0 (0)	65	0	0	0	58 (96.7)	2	7	12	39	56 (100)	0	20	21	15	9.3*	9.5*
Claudin 4	0 (0)	65	0	0	0	57 (95)	3	2	10	45	55 (98.2)	1	3	34	18	9.5*	9.2*
TTF-1	0 (0)	65	0	0	0	54 (90)	6	2	6	46	5 (8.9)	51	5	0	0	8.7*	1.01
Napsin-A	0 (0)	65	0	0	0	48 (80)	12	9	3	36	2 (3.6)	54	2	0	0	7.7*	0.34
MOC-31	8 (12.3)	57	5	2	1	55 (91.7)	5	9	8	38	51 (91.1)	5	8	18	25	8.3*	7.8*
P63	15 (23.1)	50	11	3	1	32 (53.3)	28	16	10	6	56 (100)	0	1	2	53	3.2	9.0*
CK5/6	45 (69.2)	20	8	11	26	13 (21.7)	47	6	5	2	55 (98.2)	1	1	5	49	5.2*	4.6*
P40	3 (4.6)	62	3	0	0	6 (10)	54	5	1	0	55 (98.2)	1	0	3	52	0.52	9.1*

WT1: Wilm’s tumor 1; CEA: carcinoembryonic antigen; TTF-1: thyroid transcription factor-1; CK: cytokeratin.

Immunohistochemical score 0: 0% positive cells or trace staining; 1+: 1–10% positive cells; 2+: 11–50% positive cells; 3+: >51% positive cells! The Z-score is calculated by the MannWhitney ‘U’ test between immunohistochemical scores of epithelioid mesothelioma and lung cancer (adenocarcinoma or squamous cell carcinoma).

EM: epithelioid mesothelioma; LAC: lung adenocarcinoma; LSqCC: lung squamous cell carcinoma *significant p-value (<0.0001).

### MUC4 expression

**Figure 1:**
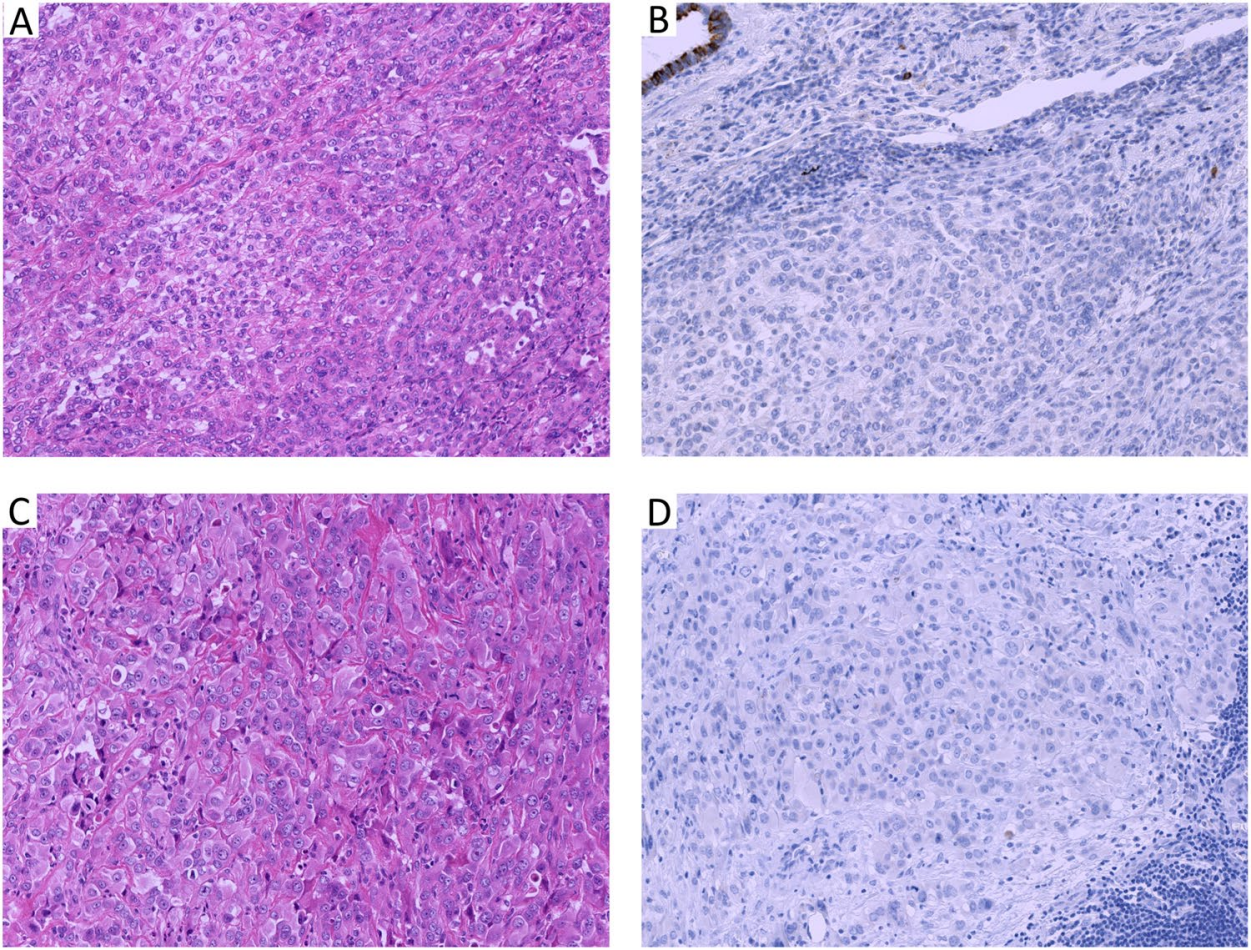
Representative cases of epithelioid mesothelioma with papillo-tubular (**A**) and solid (**C**) growth showing no MUC4 expression (**B**,**D**). Note: normal bronchial epithelium as internal control showing MUC4 expression. (**A**,**C)** H & E stain × 40 high power field; (**B**,**D)** MUC4 immunohistochemistry).

**Figure 2:**
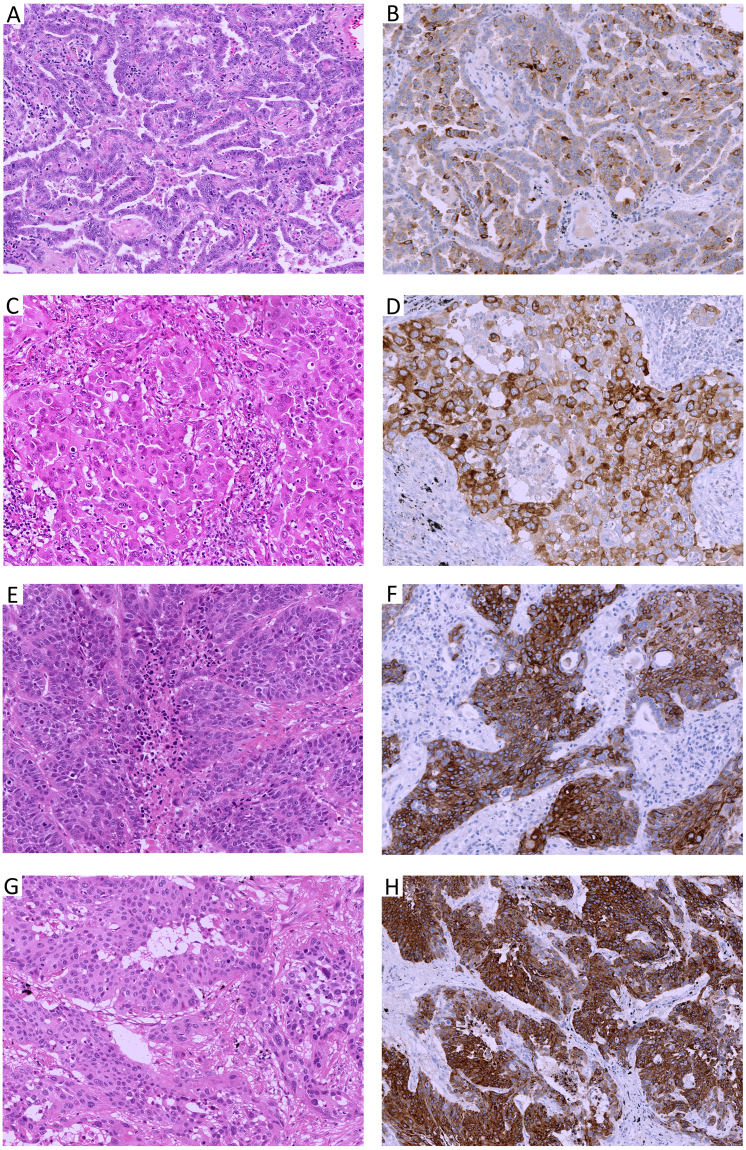
Representative cases of lung adenocarcinomas, papillary (2 A) or solid (2 C) growth and non-keratinizing squamous cell carcinoma (**E**,**G**) showing MUC4 expression. (**A**,**C**,**E**,**G)**: H & E stain × 40 high power field, (**B**,**D**,**F**,**H)**: MUC4 immunohistochemistry).

MUC4 expression was not present in any of 65 epithelioid mesotheliomas (0%, Fig. 1B,D). MUC4 expression was present in the cytoplasm of the tumor cell of lung adenocarcinoma and squamous cell carcinoma. It was also evident in the normal bronchial epithelium, considered as an internal positive control. It was present in 50 of 60 cases of adenocarcinoma (83.3%, Fig. 2B,D) and 50/56 cases of squamous cell carcinoma (89.3%, Fig. 2F,H). Among lung adenocarcinoma cases, 21 cases showed immunohistochemical score 3+, 9 cases in score 2+, and 20 cases in score 1+. In lung squamous cell carcinoma, 21 cases showed score 3+, 10 cases score 2+, and 19 cases score 1+.

In addition, we found no MUC4 expression in mesothelial layer of visceral pleura present in 39 lung cancer cases.

### Calretinin, D2–40, WT1, and CK5/6

Calretinin expression was present in 64 cases (98.5%) of epithelioid mesothelioma with the immunohistochemical score of 3+ in 57. Calretinin expression was also present in 17 of 60 (28.3%) cases of lung adenocarcinoma, and 28 of 56 (50%) cases of squamous cell carcinoma. The immunohistochemical of 3+ was not present in adenocarcinoma but was present in 5 cases of squamous cell carcinoma. D2–40 expression was present in 63 cases (96.9%) of epithelioid mesothelioma with the immunohistochemical score of 3+ in 54 cases. D2–40 expression was also present in 7 of 60 (11.7%) cases of lung adenocarcinoma and 34 of 56 (60.7%) cases of squamous cell carcinoma. The immunohistochemical score of 3+ was not present in adenocarcinoma but was present in 9 cases of squamous cell carcinoma. WT1 expression was present in 56 (86.2%) epithelioid mesothelioma, with the immunohistochemical score of 3+ in 32 cases. WT expression was not present in any of the 60 adenocarcinomas but was present in 2 of 56 (3.6%) squamous cell carcinoma. CK5/6 expression was present in 45 of 65 (69.2%) epithelioid mesothelioma, and 55 of 56 (98.2%) squamous cell carcinoma, and 13 of 60 (21.7%) adenocarcinoma. The majority of epithelioid mesothelioma and squamous cell carcinoma showed the immunohistochemical score of 3+ while in adenocarcinoma showed the immunohistochemical score of 1+ to 3+.

### CEA, Claudin 4, MOC-31

CEA and Claudin 4 expressions were not present in any of the epithelioid mesotheliomas. In contrast, CEA Expression was present in both 58 (96.7%) cases of lung adenocarcinoma and 56 (100%) cases of squamous cell carcinoma. The majority of lung adenocarcinoma showed the immunohistochemical score of 3+, and only one-fourth of CEA-positive lung squamous cell carcinoma showed the immunohistochemical score of 3+.

Membranous Claudin 4 expression was present in 57 (95%) cases of adenocarcinoma with 39 cases showing the immunohistochemical score of 3+ and 55 (98.2%) cases of squamous cell carcinoma with 34 cases showing the immunohistochemical score of 2+.

MOC-31 expression was present in 55 (91.7%) and 51 (91.1%) cases of adenocarcinoma and squamous cell carcinoma. However, MOC-31 was also expressed in 8 (12.3%) epithelioid mesothelioma, of which, 5 cases showed the immunohistochemical score of 1+, 2 cases showed 2+, and 1 case showed 3+.

### TTF-1, Napsin-A, p63, p40

Nuclear expression of TTF-1 and cytoplasmic expression of Napsin-A are the positive immunohistochemical markers of lung adenocarcinoma. TTF-1 and Napsin-A expression were not present in any of 65 (0%) cases of epithelioid mesothelioma and only 54 (90%) cases and 48 (80%) cases of lung adenocarcinoma. The immunohistochemical score of 3+ for TTF-1 and Napsin-A was observed in 46 and 36 cases respectively. TTF-1 expression was also present in 5 (8.9%) cases, and Napsin-A expression in 2 (3.6%) cases of lung squamous cell carcinoma but with the immunohistochemical score of 1+.

Nuclear p63 expression and recently nuclear p40 expression are the positive immunohistochemical markers of lung squamous cell carcinoma. However, we found p63 and p40 expression in 15 (23%) and 3 (4.6%) cases of epithelioid mesothelioma although the immunohistochemical score was 1+. The p63 and p40 expression were present in 56 (100%), 55(98.2%) cases of squamous cell carcinoma and most of them with the immunohistochemical score of 3+. However, 32 (53.3%) and 6 (10%) cases of adenocarcinoma also showed immunoreactivity for p63 and p40 respectively but with the immunohistochemical score of 1+.

### Sensitivity, Specificity and accuracy rate of immunohistochemical markers

Table 3 shows the sensitivities, specificities, and accuracy rates of the immunohistochemical markers to differentiate epithelioid Mesothelioma from lung adenocarcinoma and squamous cell carcinoma. Among the negative mesothelioma markers, MUC4, CEA, Claudin 4, TTF-1, and Napsin-A showed 100% sensitivity, p40 showed 95.4%, P63 showed 76.9%, and CK5/6 showed 69.2%. When the histological type was known as lung adenocarcinoma or squamous cell carcinoma, the specificity of TTF-1, Napsin-A and p40 were high, but with combined lung adenocarcinoma and squamous cell carcinoma, it remains around or less than 50%. Among the positive mesothelioma markers, WT1 showed the highest specificity of 98.6%, but sensitivity was limited to 86.2%. The sensitivities of calretinin and D2–40 were high with 98.5% and 96.9% respectively, but the specificities were low with 61.2%and 64.7% specificity. Among the negative mesothelial markers, CEA has the highest accuracy rate of 98.9% followed by Claudin 4 of 97.8%. The accuracy of MUC4 is 91.2% which is better than that of MOC-31.

**Table 3:** Sensitivity, Specificity, and Accuracy Rate of Immunohistochemical Markers.

Antibody	Sensitivity, %	Specificity, %	Accuracy Rate, %	*p*-value*
A. Differentiation of Epithelioid Mesothelioma from Lung Cancer including Adenocarcinoma and Squamous Cell Carcinoma				
MUC4(−)	100	86.2	91.2	<0.0001
Calretinin(**+**)	98.5	61.2	74.6	<0.0001
D2–40(**+**)	96.92	64.7	76.2	<0.0001
WT1(**+**)	86.15	98.6	94.8	<0.0001
CK5/6(**+**)	69.2	41.4	51.4	0.2
CEA(−)	100	98.3	98.9	<0.0001
Claudin 4(−)	100	96.6	97.8	<0.0001
MOC31(−)	87.7	91.4	90	<0.0001
TTF-1(−)	100	50.9	68.5	<0.0001
NapsinA(−)	100	43.1	63.5	<0.0001
P63(−)	76.9	75.2	76.2	<0.0001
P40(−)	95.4	52.6	68	<0.0001
B. Differentiation of Epithelioid Mesothelioma from Lung Adenocarcinoma				
MUC4(−)	100	83.3	92	<0.0001
Calretinin(**+**)	71.7	98.5	85.6	<0.0001
D2–40(**+**)	88.3	96.9	92.8	<0.0001
WT1(**+**)	100	86.2	92.8	<0.0001
CK5/6(**+**)	69.2	78.3	73.6	<0.0001
CEA(−)	100	96.7	98.4	<0.0001
Claudin 4(−)	100	95	97.6	<0.0001
MOC31(−)	87.7	91.7	89.6	<0.0001
TTF-1(−)	100	90	95.2	0.02
NapsinA(−)	100	80	90.4	0.21
P63(−)	76.9	53.3	65.6	<0.0001
P40(−)	95.4	10	54.4	<0.0001
C. Differentiation of Epithelioid Mesothelioma from Lung Squamous Cell Carcinoma				
MUC4(−)	100	89.3	95	<0.0001
Calretinin(**+**)	98.5	50	76	<0.0001
D2–40(**+**)	97	39.3	70.2	<0.0001
WT1(**+**)	86.2	96.4	91	<0.0001
CK5/6(**+**)	69.2	1.8	38	<0.0001
CEA(−)	100	100	100	<0.0001
Claudin 4(−)	100	98.2	99.2	<0.0001
MOC31(−)	87.7	91.1	89.3	<0.0001
TTF-1(−)	100	8.9	57.9	<0.0001
NapsinA(−)	100	3.6	55.4	<0.0001
P63(−)	76.9	100	88.5	0.008
P40(−)	95.4	98.2	96.7	0.31

WT1: Wilm’s tumor 1; CEA: carcinoembryonic antigen; TTF-1: thyroid transcription factor-1; CK: cytokeratin.

*p-value is calculated by Fisher’s exact test.

## Discussion

MUC4 is a transmembrane mucin expressed in normal epithelial cells including epithelial mucosa of the digestive tract, ductal epithelium of salivary gland and lacrimal gland, larynx and trachea, lung, stomach, intestine, uterus, cervix, mammary gland, ovary, and kidney^7^. Many human malignancies like carcinomas of the pancreas^8^, ovary^9^, salivary gland^10^, lung^11^, stomach^12^, breast^13^ show MUC4 expression. MUC4 expression has been reported in the non-small cell lung cancer more in adenocarcinoma than do squamous cell carcinomas and large cell carcinomas^14^. MUC4 expression was also present significantly in solid adenocarcinoma of the lung^15^. In addition to poor prognosis correlated with MUC4 expression in the biliary tract, pancreas, ovary, and colorectal junction^16^, the MUC4 mRNA expression is related to the tumor histological type and its differentiation^17^. The tubular and papillary patterns of adenocarcinomas and the nests and in squamous pearls of squamous cell carcinomas show strong membranous and less cytoplasmic expression of MUC4^18^. MUC4 plays a role in cell differentiation rather than cell proliferation of the normal goblet cells, the stratified squamous epithelial cells, and malignant epithelial tumor cells^19^. We previously reported the MUC4 expression in sarcomatoid carcinoma of the lung and its utility to differentiate from sarcomatoid mesothelioma^20^.

In this study, We found MUC4 expression in 50/60 (83.3%) cases of lung adenocarcinoma, 50/56 (89.3%) cases of squamous cell carcinoma, and none (0%) of epithelioid mesothelioma. Kwon *et al*.^14^  and Llinares *et al*.^21^  previously reported the diagnostic value of MUC4 expression in distinguishing epithelioid mesothelioma and lung adenocarcinoma. They reported the diagnostic value of MUC4 immunostaining in distinguishing mesothelioma and lung adenocarcinoma. They raised the polyclonal antibody against a KLH conjugate of a synthetic peptide corresponding to the tandem repeat sequence of MUC4 for their study. They found that MUC4 was expressed in 0 of the 41 epithelioid mesotheliomas and 32 of the 35 (91%) lung adenocarcinoma. Our study utilized the commercially available antibody and is the validation of the previous study. Other things different from this study is the inclusion of squamous cell carcinoma which also showed similar frequencies of positive cases like that of lung adenocarcinoma.

Our data of MUC4 expression in adenocarcinoma and squamous cell carcinoma of lung is also similar to another study by Kwon *et al*. who studied MUC4 expression in lung adenocarcinoma and squamous cell carcinoma of the lung but without mesothelioma.

In this study, we analyzed the utility of MUC4 to distinguish epithelioid mesothelioma from lung adenocarcinoma and squamous cell carcinoma. We have previously reported the MUC4 expression in sarcomatoid carcinoma of the lung and its utility to differentiate from sarcomatoid mesothelioma^20^. In our previous report, we emphasized the MUC4 expression in spindled cells of sarcomatoid carcinoma of lung. None of sarcomatoid mesothelioma had the expression of MUC4 expression. MUC4 was expressed in 72% of the sarcomatoid carcinoma of lung.

Ordonez *et al*.^22^ reported that there is no absolute specific and sensitive marker of epithelioid mesothelioma, although the various immunohistochemical markers are currently available for the diagnosis of epithelioid mesotheliomas. He also added the location and histologic features of the tumor, the sex of the patient, and the clinical findings need to be considered when selecting the markers for accurate diagnosis of epithelioid mesothelioma from the various other carcinomas^22^.

The sensitivity and specificity of MUC4 to differentiate epithelioid mesothelioma from lung adenocarcinoma were 100% and 83.3% respectively with the accuracy rate of 92% and those of MUC4 to differentiate epithelioid mesothelioma from lung squamous cell carcinoma 100% and 89.3% respectively with the accuracy rate of 95%. We found CEA is the best marker with the sensitivity of 100%, specificity of 98.3%, and accuracy rate of 98.9% followed by Claudin 4 which had the sensitivity of 100%, specificity of 96.6%, and accuracy rate of 97.8%. MUC4 expression showed a similar sensitivity of 100%, but the lower specificity of 86.2% and accuracy rate of 91.2%. However, it showed better sensitivity, specificity and accuracy rate than that of MOC-31. Also, we found positive immunoreactivity for MUC4 in some cases of adenocarcinoma and squamous cell carcinoma with no CEA and/or Claudin 4 expression. Two cases of CEA-negative adenocarcinoma, one of which was negative for Claudin 4 too, showed MUC4 expression (immunohistochemical score of 2+ or 3+). Moreover, 3 cases of Claudin 4-negative adenocarcinoma, two of which were negative for CEA too, showed MUC4 expression (immunohistochemical score of 1+ or 2+). There was one adenocarcinoma case which was negative for both CEA and Claudin-4 but positive MUC4 expression. So, MUC4 has the potential for the use as an additional negative marker of epithelioid mesothelioma for differentiation from lung cancer including adenocarcinoma or squamous cell carcinoma.

International Mesothelioma Interest Group Guideline for pathologic diagnosis of malignant mesothelioma has recommended various immunohistochemical negative markers, CEA, MOC-31, TTF-1, Napsin-A to differentiate it from adenocarcinoma and p40, p63, MOC-31 to differentiate it from squamous cell carcinoma^4^. Ordonez *et al*. later added Claudin 4 as one of the best broad-spectrum carcinoma markers to discriminate epithelioid mesotheliomas from both lung adenocarcinomas and squamous cell carcinomas^23^. In our previous publication too, we found CEA and Claudin 4 were the best positive carcinoma markers for discriminating epithelioid mesotheliomas from squamous cell carcinomas^24^. Although p63 is the marker of squamous cell carcinoma, it was at least focally positive in many of adenocarcinoma (32/60, 53.3%) and epithelioid mesothelioma (15/65, 23.1%) in the present study. So, limiting the value of the p63 expression for differentiation of epithelioid mesothelioma from squamous cell carcinoma.

In conclusion, MUC4 can be added as additional positive marker of non-small cell carcinoma (both lung adenocarcinoma and squamous cell carcinoma) and negative immunohistochemical marker to differentiate epithelioid mesothelioma from lung adenocarcinoma and squamous cell carcinoma. This study includes the limited cases (65 epithelioid mesothelioma and 116 cases of lung adenocarcinoma and needs follow up study consisting of large numbers of cases.
